# Mapping lower secondary school students’ conceptions of three aspects critical for understanding the nervous system

**DOI:** 10.1371/journal.pone.0301090

**Published:** 2024-05-06

**Authors:** Pål Kvello

**Affiliations:** Department of Teacher Education, Norwegian University of Science and Technology, Trondheim, Norway; NTNU: National Taiwan Normal University, TAIWAN

## Abstract

Understanding the nervous system is an important but perhaps ambitious goal, particularly for students in lower secondary education. It is important because of its’ direct role in both mental and physical health, and it is ambitious because instruction focuses on the human nervous system, which is extremely complex, and subject to numerous misconceptions. Despite its’ complexity, the science curricula, both nationally and internationally, emphasize an understanding of the system, and not just knowledge of isolated facts. But what does it mean to understand this system, and what content knowledge is critical for understanding it? Unfortunately, the curricula are usually too general to answer these questions, therefore other sources of information are needed. Using the science literature, the present study defines the system level of the nervous system and proposes three basic aspects necessary to understand it: 1) neural circuit architecture, 2) synaptic action, and 3) nerve signal origin. With this background, the aim of the present study is to identify lower secondary school students’ conceptions of these three aspects, and to determine how they impact students’ understanding of the system. To reach this aim, the study used a questionary which allowed for a mixed method design, and the results show that many students have an immediate conception of the brain as the origin of nerve signals. In addition, many students hold the alternative conceptions that 1) synaptic action is exclusively excitatory, and that 2) neural circuits consists of neurons connected in a chain, one single neuron after another. These alternative conceptions prevent students from understanding the system. Implications for instruction are discussed in the context of conceptual learning theories, and teaching strategies are proposed. Since similar curricula goals and textbook content exist in several countries, the present results may be representative across nations.

## Introduction

Understanding the nervous system is an important but perhaps ambitious curriculum goal, particularly for students in lower secondary school. It is important because of its’ direct role in both mental and physical health [1], and it is ambitious because teachers often focus on the human nervous system which is subject to numerous misconceptions [2–4] and extremely complex. With billions of neurons [5], each communicating at a speed of up to 120 m/s [6], by passing nerve signals through thousands of synapses [7] which may increase or decrease in strength [8] within a few seconds [9], the nervous system generates our perceptions, feelings, thoughts, and behaviors, and it allows us to learn the present, remember the past, and predict the future. Despite this complexity, the science curricula of these young students, both nationally and internationally, emphasize an understanding of the system, and not just knowledge of isolated facts [10–12]. But what does it mean to understand the nervous system whose complexity is far beyond its’ physiological counterparts, and what content knowledge is critical for understanding it? Unfortunately, the curricula are usually too general to answer these questions, therefore other sources of information are needed. According to [13,14], the nervous system can be understood at different levels, and one of these levels is, in fact, called the system level. An understanding at this level pertains to the function of neural circuits, and it describes how nerve signals travelling in circuits of neurons can produce specific functions in the organism. Content knowledge critical for understanding this level of the nervous system can be derived from the systems thinking approach of Arnold and Wade [15]. From a literature study, these authors describe eight elements which are important for understanding any system. However, the first and most basic element are the key connections between parts of the system. In the nervous system, this corresponds to the aspect of how neurons are connected in a circuit (neural circuit architecture), and how connected neurons impact each other (synaptic action). Another element are the types of flows in the system. In the nervous system, this corresponds to the aspect of nerve signals and where they start (nerve signal origin). Consequently, to understand the nervous system, one would expect students to have scientific conceptions of these three aspects. But what are these scientific conceptions? According to the literature, neural circuit architecture has been described as a chain [16] and as a network with divergence and/or convergence [17], but only the network with both divergence and convergence is considered scientifically correct [1, principle 6b and c]. Synaptic action has been described as excitatory, inhibitory [18], and modulatory [19], and the nerve signals are found to originate from the senses [e.g., 20] and from the brain [e.g., 21]. The importance of these conceptions for secondary science education is emphasized by a recent Delphi-study on the nervous system [1, principle 4, 6, 7b, 11–13]. However, the study does not distinguish between lower and upper secondary education. Therefore, the question is whether students in lower secondary have these scientific conceptions? In lower secondary education, the three aspects (neural circuit architecture, synaptic action, and nerve signal origin) are often taught in context of the knee-jerk reflex or the withdrawal reflex. Unfortunately, many textbooks present these reflexes by simplified models which may lead to misconceptions. For example, neural circuit architecture is usually presented as a chain of neurons, connected one single neuron after another. Synaptic action is usually presented as being exclusively excitatory, and the nerve signals are usually presented as originating exclusively from the senses [e.g., 22–25]. Since students’ conceptions often can be traced back to the literature [26,27], there is reason to believe that lower secondary school students complete formal education on the nervous system with misconceptions of the three aspects. If this is true, then they are unlikely to understand the system, and current science education must be changed. However, this needs to be verified. Therefore, the aim of the present study is to identify lower secondary school students’ conceptions of 1) neural circuit architecture, 2) synaptic action, and 3) nerve signal origin, and determine how these conceptions may impact their understanding of the system. Implications for teaching are discussed in relation to three theories on conceptual learning.

### Students’ conception of the nervous system

Although there are many old and recent studies investigating people’s conceptions of the nervous system, these studies mainly focus on the brain at the psychological level [e.g., 3,28]. Only a few studies have investigated peoples’ conceptions of the nervous system at the anatomical and physiological level consistent with basic school curricula. These studies show that children down to the age of four know that the brain is located in the head [29,30]. However, they seem to think that the brain is independent from the rest of the body. By third grade (8–9 years) most children know that the brain is connected to other parts of the body, and by ninth grade (14–15 years) most students seem to know that this connection is reciprocal. Constituents of the nervous system like nerves and cells are familiar to some fifth and sixth graders, but in general, knowledge about the structure and functioning of the nervous system is low [30]. Consequently, it is reasonable to assume that there is little understanding of the system level at these lower grades. Neither is it expected from the curricula. In higher grades, however, the curricula emphasize an understanding of the system. For example, in eight–tenth grade (lower secondary education), English students should learn about the structure–function relationships of the human nervous system [10], Norwegian students should be able to describe how drugs, medicine, environmental toxins, and doping influence the nervous system [12], and in the USA, systems understanding is a crosscutting concept with the nervous system as one of several systems to be learned [11]. The crosscutting concepts at this grade even specify that learning about connections of components in a system, as well as the information flow both within and between systems, are important. This corresponds well with the three aspects to be investigated in the present study. Despite several years with this political emphasis on understanding the system, no studies have yet investigated lower secondary school students’ understanding of the nervous system at the system level. Therefore, this is the first study trying to clarify what an understanding of the nervous system means in lower secondary education, as well as trying to identify the current state among students. Contextualized by theories on conceptual learning, the results from the present study may provide a basis from which new teaching approaches can start to develop for the nervous system in lower secondary education.

### Learning of scientific concepts

Learning of science begins already when children start interacting with their environment. Therefore, when beginning formal education, students usually have prior knowledge about the scientific concepts to be taught. According to the constructivist view, this knowledge is used by students to understand new scientific concepts [31]. When prior knowledge is consistent with the new scientific concepts, learning is easy and takes place through the process of assimilation. According to [32], this is a process where new scientific concepts are adapted to the student’s pre-existing mental schema (prior knowledge). However, if the prior knowledge is inconsistent with the new scientific concepts, students are said to have developed misconceptions, and learning often becomes more difficult. According to [33], a misconception is defined as an understanding or explanation that differs from what is known to be scientifically correct. In addition, most uses of the term include that the incorrect understanding must also be persistent and commonly held (prevalent) [34]. Unfortunately, the term misconception has become problematic for several reasons, and other terms have been proposed, albeit without consensus [35]. Therefore, from this point on, the present study will use the term Alternative conception to denote the definition above. Due to the learning challenges usually associated with alternative conceptions, they have received much attention by the education research community. A major goal has been to develop a theory which can describe the structure and development of knowledge, and link specific types of alternative conceptions to specific types of instructional designs [36,37]. Although no such comprehensive theory currently exists, important steps have been made, for example by the Framework theory [38], the Knowledge-in-Pieces theory [36], and more recently, the Domain of Validity theory [39].

### The framework theory

According to [38] framework theory, children’s early knowledge system consists of loosely interrelated conceptions organized as a relatively stable and relatively coherent conceptual (theory-like) framework. Children use these conceptions to explain scientific phenomena and form predictions. However, since these conceptions are usually different from the scientific ones, childrens explanations and predictions are sometimes incorrect. This can be corrected through learning, but it is difficult because the relatively stable and coherent structure of knowledge proposed by this theory, requires radical changes in students’ ontology and epistemology. Therefore, with learning, this theory claims that childrens’ knowledge system develops slowly towards scientific conceptions through three major types of alternative conceptions. One is called “initial conceptions”, and it is developed from everyday experiences before children have been exposed to school science [37]. The two others are called “synthetic conceptions” and “fragmented conceptions”, both of which are developed after students have been exposed to school science. Although both are erroneous, the synthetic conceptions have some explanatory power and internal consistency, whereas the fragmented conceptions lack these properties [38]. They are formed when students try to reconcile initial conceptions (which are incorrect) with new scientific knowledge, without adequate instruction. Inaccurate language and illustrations in textbooks are pointed out as some potential causes [37]. Therefore, it is important that instruction and curricula provide explanations and models that are accurate, and that all the information necessary to restructure students’ initial ontology and epistemology is included. This should be complemented with instruction that focus on the deep exploration of a few key concepts instead of covering a great deal of material in a superficial way, and particular attention should be paid to the alternative conceptions that are persistent, since they may constrain learning [37].

### The knowledge-in-pieces theory

Another theory has been proposed by [36]. According to this theory called Knowledge in Pieces (KiP), children’s early knowledge system consists of small, relatively isolated and self-explanatory knowledge elements called phenomenological primitives (P-prims). They seem to originate from superficial interpretations of experienced reality, not school science, and they provide a “sense of mechanism” for how things work, and therefore a sense of casualty [40]. P-prims appear in peoples’ mind rather spontaneously, and often unconsciously, in a delicate context-dependent manner to help explain scientific phenomena and form predictions. One example is the Ohm’s p-prim which says something like: “increased effort leads to more result”. In some contexts, this is true, in others, it is not. Hence, p-prims are different from scientific concepts, they only work in some contexts but fail in others. Nevertheless, p-prims are not considered obstacles to learning but rather as resources from which scientific conceptions can be built. Through learning, this theory claims that childrens’ knowledge system develops towards scientific conceptions through collecting and systematizing p-prims into larger wholes. During this process, p-prims change their function from relatively isolated, self-explanatory elements to become integrated in a larger system of complex knowledge structures such as physics laws. Since p-prims appear in a variety of contexts, but only work when used inside their range of legitimate applicability [40], this theory suggests that instruction should expose students to multiple contexts, while encouraging student reflection under guidance. In this way, students may find contexts where the p-prims are productive and where they are not. Since the KiP theory and the framework theory explain knowledge structure at different levels of resolution (p-prims are sometimes considered building blocks of concepts), they are not mutually exclusive, but may rather be considered complementary.

### The domain of validity theory

Since the KiP theory and the Framework theory both focus on knowledge structure and development, a third theory, proposed by [39] provides an important extension by focusing more on instructional design. According to their Domain of Validity theory (DoV), knowledge consists of two connected elements: a model and a domain of validity, i. e. a concept and a context. Students’ conceptions are usually applied in a context, and even though the applied conceptions may be different from the scientific conceptions, they may still explain certain phenomena successfully. [39] claims that this is the reason many alternative conceptions exist–because they have been applied successfully to explain some scientific phenomena in the past. They become a problem only when they are applied in a new context where they no longer are appropriate, i. e. they are used beyond their domain of validity. In such cases, the concept has obtained an Overgeneralized Domain of Validity (ODoV). According to this theory, instructional design should focus on reducing the concept’s domain of validity, and this can be achieved by creating a cognitive conflict which makes both the domain and the concept explicit to the students. This theory emphasizes the importance of making both the concept and the domain explicit to the students. Mapping conceptions and their appropriate contexts are important because it allows teachers to pinpoint precisely the cognitive conflict that students need to confront in a conceptual change approach to teaching.

## Materials and methods

### Study design

The present study was designed with the purpose of collecting information about student’s conceptions of three specific aspects of the nervous system, and to determine how these conceptions would influence their understanding of the system. It has a mixed method design using a questionary with open-ended and closed-ended items developed particularly for this study. The study was approved by the Institutional Review Board at the Department of Teacher Education (Head of Department and Deputy Head of Research) at the Norwegian University of Science and Technology.

### Participants

The sample consisted of 229 students from eleven classes at four lower secondary schools in Norway. 127 students were from grade nine (13–15 years old) and 102 students were from grade ten (14–16 years old). All classes had completed the content about the nervous system for compulsory education to reach a common curriculum goal which says that students should be able to describe the nervous system and explain how it controls processes in the body [41]. The language of instruction was Norwegian, and the duration of a typical instructional session is 45–90 minutes. However, the specific content and instructional strategies experienced by the participants are not known.

### Questionary

**Fig 1:**
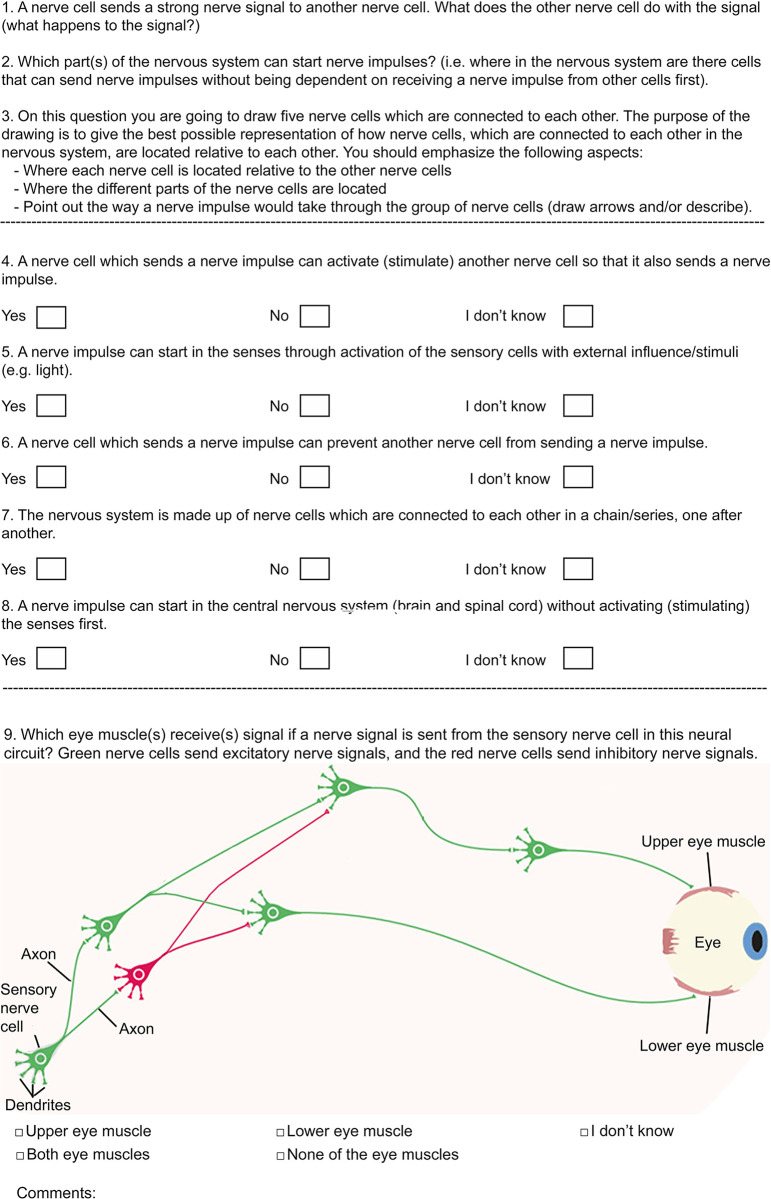
The questionary used to investigate students’ conceptions of the nervous system.

To identify students’ conceptions of neural circuit architecture, synaptic action, and nerve signal origin, and to determine how these conceptions may influence their understanding of the system, a questionary was developed consisting of nine items (Fig 1). The details on each aspect are described below.

What conceptions do students have of neural circuit architecture?

To identify students’ conceptions of neural circuit architecture, two items were developed: One open-ended question (questions 3), and one closed-ended question (question 7) (Fig 1). The open-ended question was important to obtain an initial response without providing any cues to the correct answer. It asked the students to make a drawing demonstrating how neurons are connected to each other in the nervous system (see Fig 1 for details). The question was supplemented with specific instructions which is important to stimulate drawings of high quality [42,43]. In line with [44], drawing was used as the response format because the knowledge involved specific anatomy which is not easily represented explicitly via descriptive explanations. The closed-ended question asked for students’ responses to the assertion that the nervous system is made up of neurons connected in a chain, one after another. This was important to directly confront students with the potential alternative conception identified in the open-ended question. The assertion was followed by the three single-select response alternatives “yes”, “no” and “I do not know” to provide a minimum of potential cues to the correct answer which is a common concern of multiple-choice tests [45].

What conceptions do students have of synaptic action?

To identify students’ conceptions of synaptic action, three items were developed: One open-ended question (question 1), and two closed-ended questions (question 4 and 6). Again, the open-ended question was important to obtain an initial response without providing any cues to the correct answer. It asked the students what a nerve cell, which receives a strong nerve signal, does with the signal (see Fig 1 for details). The response format was not specified, but lines were included below the question to encouraged writing because the knowledge involved a process which is not easily represented explicitly via figurative explanations. The first closed-ended question asked for students’ responses to the assertion that a neuron, which sends a nerve impulse, can stimulate another neuron to send a nerve impulse (question 4). The second closed-ended question asked for students’ responses to the assertion that a neuron, which sends a nerve impulse, can prevent another neuron from sending a nerve impulse (question 6). Both were important to directly confront students with the potential alternative conception identified in the open-ended questions. The assertion was followed by the three single-select response alternatives “yes”, “no” and “I do not know” to provide a minimum of potential cues to the correct answer which is a common concern of multiple-choice tests [45].

What conceptions do students have of nerve signal origin?

To identify students’ conceptions of nerve signal origin, three items were developed: One open-ended question (question 2), and two closed-ended questions (question 5 and 8). Again, the open-ended question was important to obtain an initial response without providing any cues to the correct answer. It asked the students about which part(s) of the nervous system can start nerve impulses independent of other nerve cells (see Fig 1 for details). The response format was not specified, but lines were included below the question to encouraged writing. The first closed-ended question (question 5) asked for students’ responses to the assertion that a nerve impulse can start within the senses by stimulating the sensory cells with external stimuli. The second closed-ended question asked for students’ responses to the assertion that a nerve impulse can start in the central nervous system (brain and spinal cord) without activating the senses first (question 8). Again, both were important to directly confront students with the potential alternative conception identified in the open-ended questions, and the assertion was followed by the three single-select response alternatives “yes”, “no” and “I do not know” to provide a minimum of potential cues to the correct answer which is a common concern of multiple-choice tests [45].

How do students’ conceptions of neural circuit architecture, synaptic action, and nerve signal origin influence their understanding of the system level?

To determine how students’ conceptions of the three aspects may influence their understanding of the system level, one last item was developed. This was a closed-ended question (question 9) related to the illustration in Fig 1. It asked which eye muscle(s) receive(s) signal if a nerve signal is sent from the sensory nerve cell in this neural circuit? The question was followed by some information about excitatory and inhibitory nerve signals, and by four informative response alternatives as well as an “I don’t know” alternative to reduce the pressure of forced choice. In addition, an open-ended comment field was included to give the students a chance to justify their answers. This is comparable to a two-tier multiple-choice test [46], except that the response field in the present study was made open-ended to allow for any possible explanation instead of some forced-choice alternatives. The question was important to determine students’ understanding of the system, and the open-ended comment field was important to determine how their understanding of the system was influenced by the potential alternative conceptions.

### Development

The questionary was developed through several cycles of testing, feedback and revisions. As test candidates, relevant groups of people were used including 28 secondary school students, 29 science teacher students and three professors in science education. The professors and two of the secondary school students participated in an interview after the test. This was a structured interview which followed the questions in the questionary. The respondents were asked how they understood the questions and why they responded the way they did. Through this process three major types of changes were made: 1) Changes necessary to make the questions easier to understand, e.g., the term “stimulate” in question 4 was supplemented with the term “activate” because the former term was unknown to some of the secondary school students. Similarly, the term “action potential” was replaced by “nerve signal” and “nerve impulse”. 2) Changes to reduce biases, e.g., the phrase “network of nerve cells” was replaced by “group of nerve cells” in question 3 (drawing) since the first phrase triggered associations which biased the drawings. 3) Changes to increase attention, e.g., the neural circuit illustrated in question number 9 was changed from the traditional knee-jerk reflex circuit (including an inhibitory neuron) to the present artificial circuit because many students seemed to respond reflexively based on previous experience with a simplified knee-jerk circuit instead of actually looking into the logic of the presented neural circuit. The questionary was then updated to increase the probability that the students understood all the items, and that the necessary time to complete it was within an acceptable time range (ca. 15 min). As recommended by [47], the questionary was made as short as possible to increase the probability that students maintained their focus on the questions and completed them properly.

### Data collection

At the beginning of a regular science class, the researcher, with the science teacher present, informed the students about the research project, and told them that participation was voluntarily and anonymous. All students received the questionary, and both those who wanted to participate and those who did not want to participate could return the questionary, with or without responses, in a box placed on a table in the classroom. In this way, informed verbal consent as well as research data was obtained anonymously. Consequently, the data was obtained and handled in line with the principles expressed in the Declaration of Helsinki. The different types of questions were handed out to the students and returned in a sequential order. First, the open-ended questions were completed (questions 1–3). Second, the multiple-choice items with the response alternatives” yes”, “no” and “I do not know” were completed (questions 4–8). Third, the multiple-choice question related to the illustration was completed (question 9). The reason for this sequential order was to minimize the students’ chance of using information inherent in one type of questions to answer any of the other types. The open-ended questions were completed first because they contained the lowest amount of information, and question 9 was completed last because it contained most information. The students were not given a time limit for the completion of the questionary, but all students completed within 20 minutes. Although some students spent less time than others, differences in the duration among students were not recorded.

### Analysis

To identify potential alternative conceptions according to the definition of a misconception made by [33], students’ responses were analyzed and categorized by a combination of inductive and deductive coding [48]. For open-ended question 1, deductive coding was used to categorize students’ responses according to the three main concepts of synaptic action described in the literature: 1) the concept of excitatory synaptic action, 2) the concept of inhibitory synaptic action [18], and 3) the concept of modulatory synaptic action [19]. For open-ended question 2, students’ responses were not coded because they were short and clear, mostly consisting of single words like the brain, senses, or the spinal cord. In fact, many answers included the two concepts of nerve signal origin described in the literature: 1) the concept of sensory-origin [e.g., 20], and 2) the concept of brain-origin [e.g., 21]. Therefore, the complete answers were directly used as response categories. For open-ended question 3, deductive coding was used to categorize students’ drawings according to the four main concepts of neural circuit architecture described in the literature including: 1) open chain, 2) closed chain [16], 3) network with divergent connections, and 4) network with convergent connections [17]. In addition, inductive coding was used to establish four other categories from drawings that did not have suitable representations in the literature. The reliability of the deductive coding of students’ responses to question 1 and 3 was tested by having two science teacher students and one researcher independently coding the data, and subsequently using the Krippendorff’s alpha test [49] to estimate the inter-coder reliability. The test was conducted in the software SPSS (IBM SPSS statistics for Windows, version 27.0.) after implementing a macro downloaded from the webpage http://afhayes.com/spss-sas-and-r-macros-and-code.html. The Krippendorff’s alpha values (α) are reported in the results below. In contrast, the inductively generated categories were developed through consensus among the researcher and the two teacher students. For multiple-choice assertion 4–8, students’ responses were categorized according to the predefined response alternatives YES, NO and I DON’T KNOW. Since these categories were already defined as similar to, or different from what is known to be scientifically correct, no further analysis was needed to conclude according to the definition made by [33].

To determine whether the alternative conceptions identified from the definition above also complied with the extended definition of a misconception made by [34], both prevalence and persistence were calculated. According to the online oxford dictionary [50], prevalence is defined as the condition of being widespread, and persistence is defined as the continued existence of something. Since there is no consensus on an absolute critical value for claiming that a phenomenon is prevalent or persistent, we determined this based on relative values. Therefore, the prevalence of an alternative conception was first calculated as the percentage of students demonstrating the alternative conception on the open-ended questions, and subsequently a binomial test was used to determine if this proportion was significantly larger than expected if the students were randomly distributed among the identified conceptual categories. A p-value = < 0.05 defined the alternative conception as prevalent. The persistence of an alternative conception, however, was first calculated as the proportion of students demonstrating the alternative conception on both the open-ended question and the multiple-choice items, and a chi-square test followed by a post hoc [51] was used to determine if this proportion was significantly larger than expected by chance. A p-value = < 0.05 defined the alternative conception as persistent. In line with the recommendations of [52], the significance threshold (α-value) was not adjusted in this study because the hypothesis was specified in advance to the results of single tests, i.e., the study was only interested in the results from one particular test for each aspect.

To identify the effect of the alternative conceptions on the student’s understanding of the system level, their answers to multiple-choice question 9 were categorized according to the predefined response alternatives, and the percentage of students within each category was calculated and presented in diagrams. In addition, associated comments which provided a relevant explanation of their answers were organized in a table and interpreted to see if their multiple-choice answer was associated with a particular conception/alternative conception of the three aspects: 1) neural circuit architecture, 2) nerve signal origin, and 3) synaptic action. In this way, their correct or incorrect answers to question 9 could be related to a particular conception/alternative conception of one or more of the three aspects.

## Results

### What conceptions do students have of neural circuit architecture?

**Fig 2:**
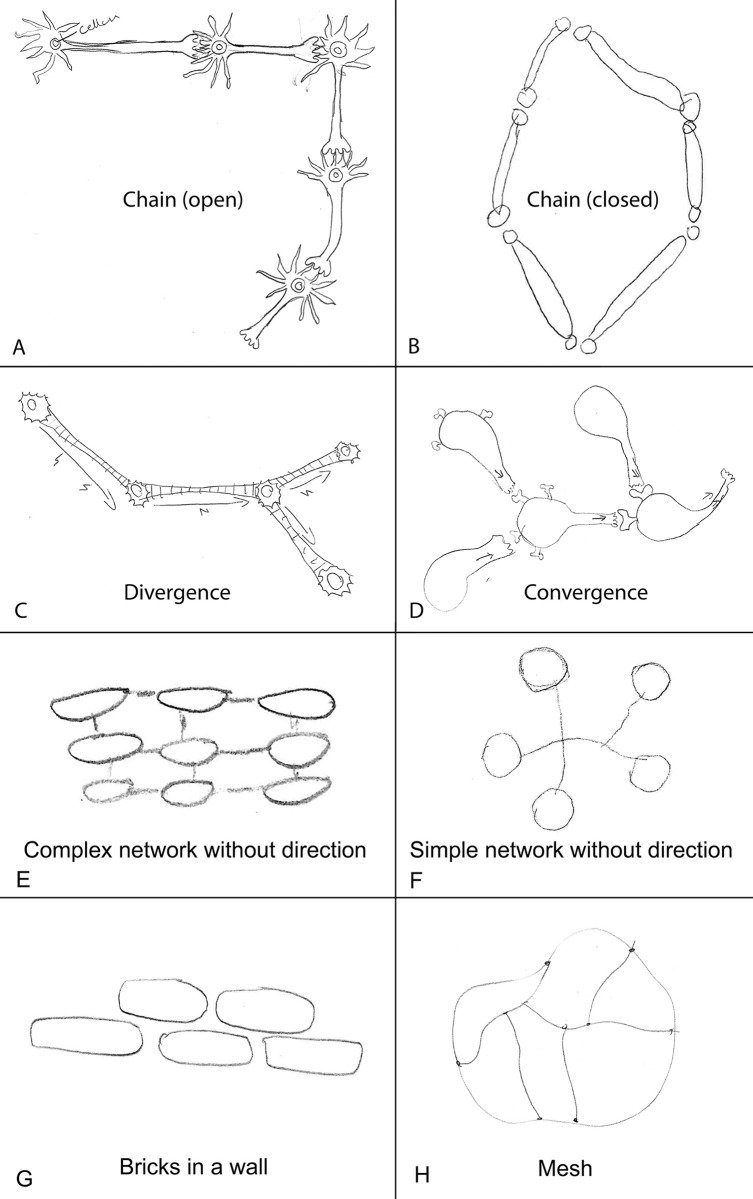
The eight conceptual categories of neural circuit architecture represented by examples from the students’ answers (drawings) on question 3 (see Fig 1 for details on question 3).

**Fig 3:**
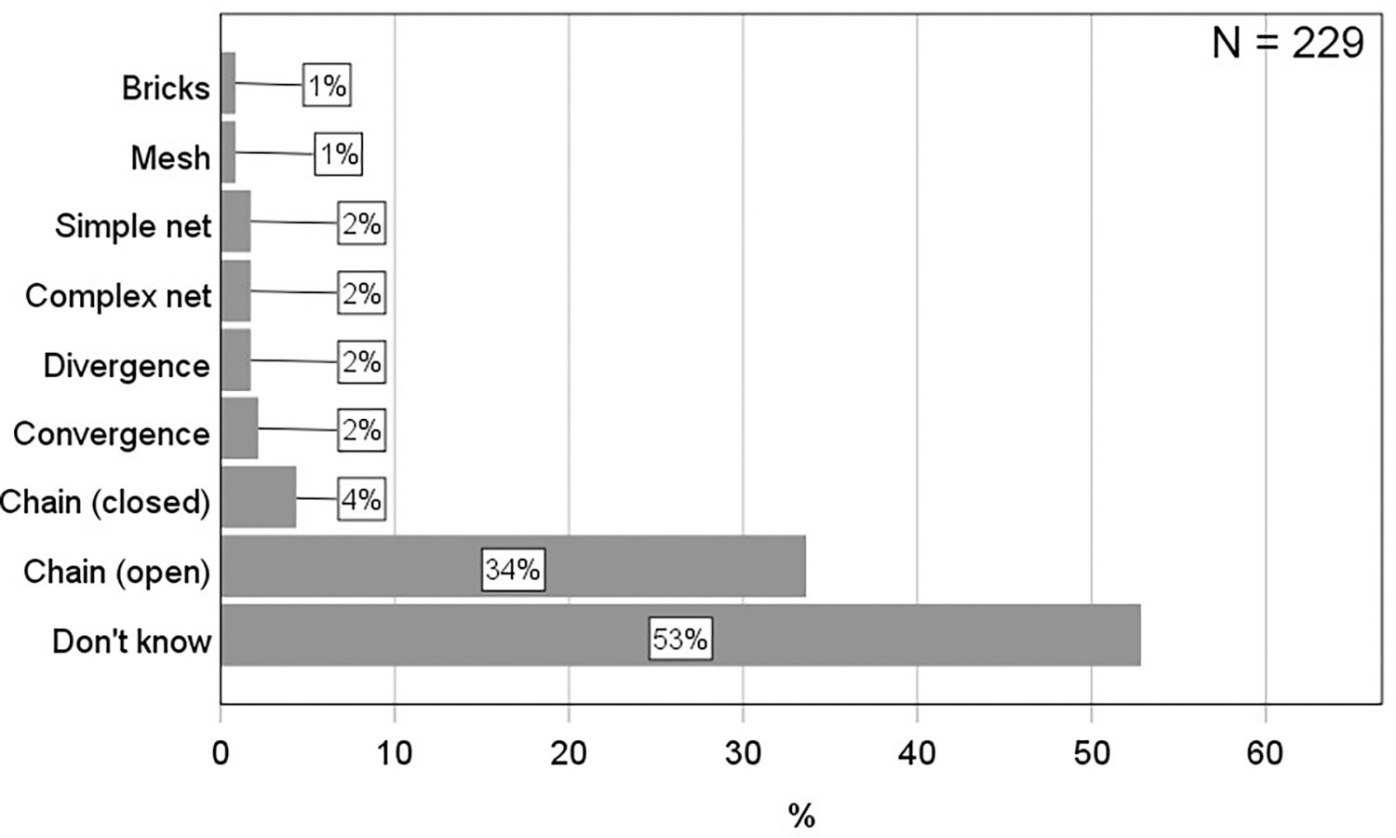
Bar graphs showing the percentage of students within each conceptual category of neural circuit architecture identified from their answers on question 3 (see Fig 1 for details on question 3).

**Fig 4:**
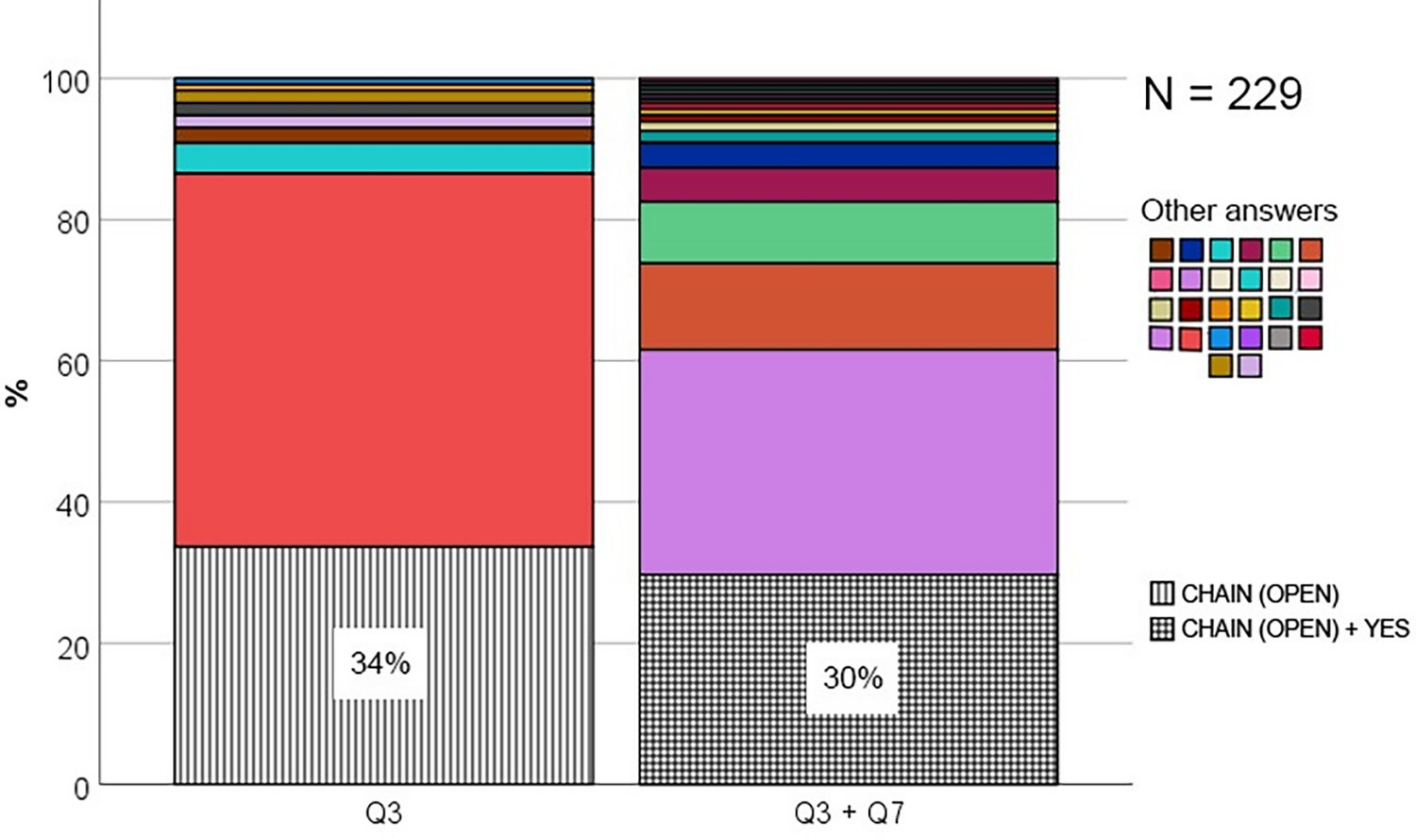
Stacked bar graphs showing the percentage of students within each conceptual category of neural circuit architecture identified from their answers on question 3 (Q3), and within each combination of answers on question 3 and 7 (Q3 + Q7). Only the category CHAIN (OPEN) is specified for Q3, and only the category YES is specified for Q7. This is because they are the only relevant categories for the purpose of demonstrating the prevalence and persistence of the Open Chain conception.

To investigate students’ conceptions of neural circuit architecture, they were initially given an open-ended question asking them to make a drawing demonstrating how neurons are connected to each other in the nervous system (see question 3 for details). This resulted in 109 relevant drawings which were categorized into eight conceptions, four of which were deductively generated and four inductively generated (Fig 2). The inter-coder reliability of the deductively generated conceptions (conceptual categories) was very high (α = 0.9134), demonstrating that the three coders did agree. To determine the prevalence of these conceptions, the percentages were calculated, showing that the most prevalent conception was the Chain conception (Fig 3). As many as 38% of the students drew neurons connected in a single chain, one neuron after another. Most of these chains were of the open type (34%), only a few were of the closed type (4%). Even fewer students made drawings which clearly included convergent (2%) or divergent (2%) connections, and no students answered correctly by including both. A similar low proportion of students (2%) made drawings of neurons connected with several continuous lines (Complex net and Simple net), but since they lacked a direction of the nerve signal, it was impossible to determine if there was convergence, divergence, or something else. A minority of students either drew neurons organized as bricks in a wall or a mesh of lines (1%). Finally, the remaining and largest proportion of students reported that they did not know how neurons are connected to each other (53%). Using a binomial test, the study showed that the only conception which was significantly more prevalent than expected from a random distribution of students among the eight identified categories (12.5%), was the Open Chain conception (34%, *p* < .001) (Fig 3 and S1 Table). Therefore, based on its’ relative prevalence, the conclusion is that the Open Chain-conception is prevalent, and it is the only prevalent conception of neural circuit architecture in this sample of students. To determine the persistence of the Open Chain conception, the students were asked a second question on this aspect. This question directly confronted them with the conception by asking whether the nervous system is made up of neurons connected in a chain, one after another (question 7). As shown in Fig 4, this was confirmed by most of the students who drew an open chain on question 3. Using a chi-square test followed by a post-hoc, the study further demonstrated that this proportion (i.e., the proportion of students drawing an open chain on question 3 and answering YES on question 7) was significantly higher than expected by chance *X*^*2*^ (1, *N* = 229) = 11.58, *p* < .001 (S2 Table). Therefore, the study conclude that the Open Chain conception is persistent over the course of two different types of questions (an open-ended question and a multiple-choice question). In summary, these results show that most students seem to lack a clear conception of neural circuit architecture. However, those who have a conception, can be grouped into eight conceptual categories among which the Open Chain conception is most prevalent. This conception also showed some persistence.

### What conceptions do students have of synaptic action?

**Fig 5:**
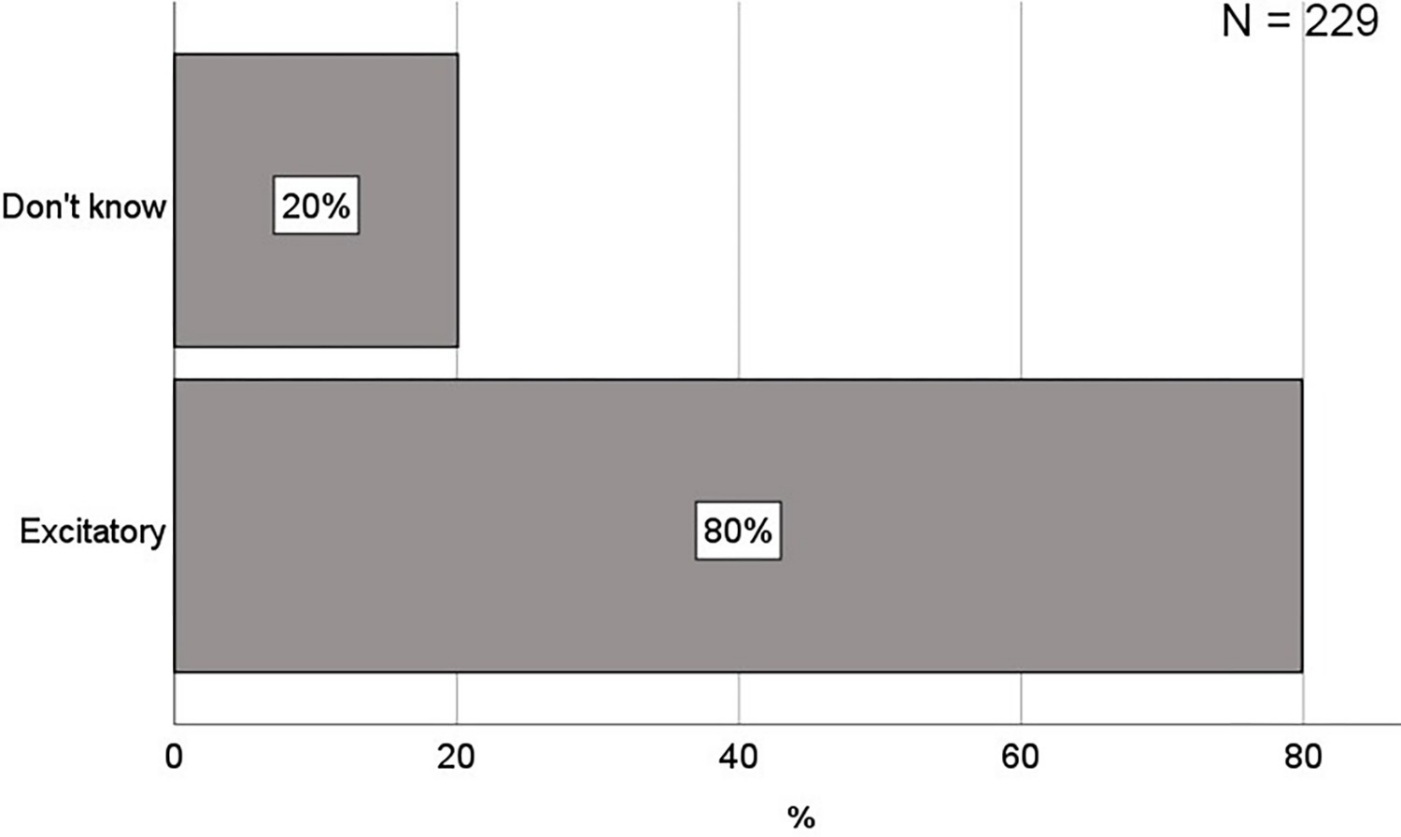
Bar graphs showing the percentage of students within each conceptual category of synaptic action identified from their answers on question 1 (see Fig 1 for details on question 1).

**Fig 6:**
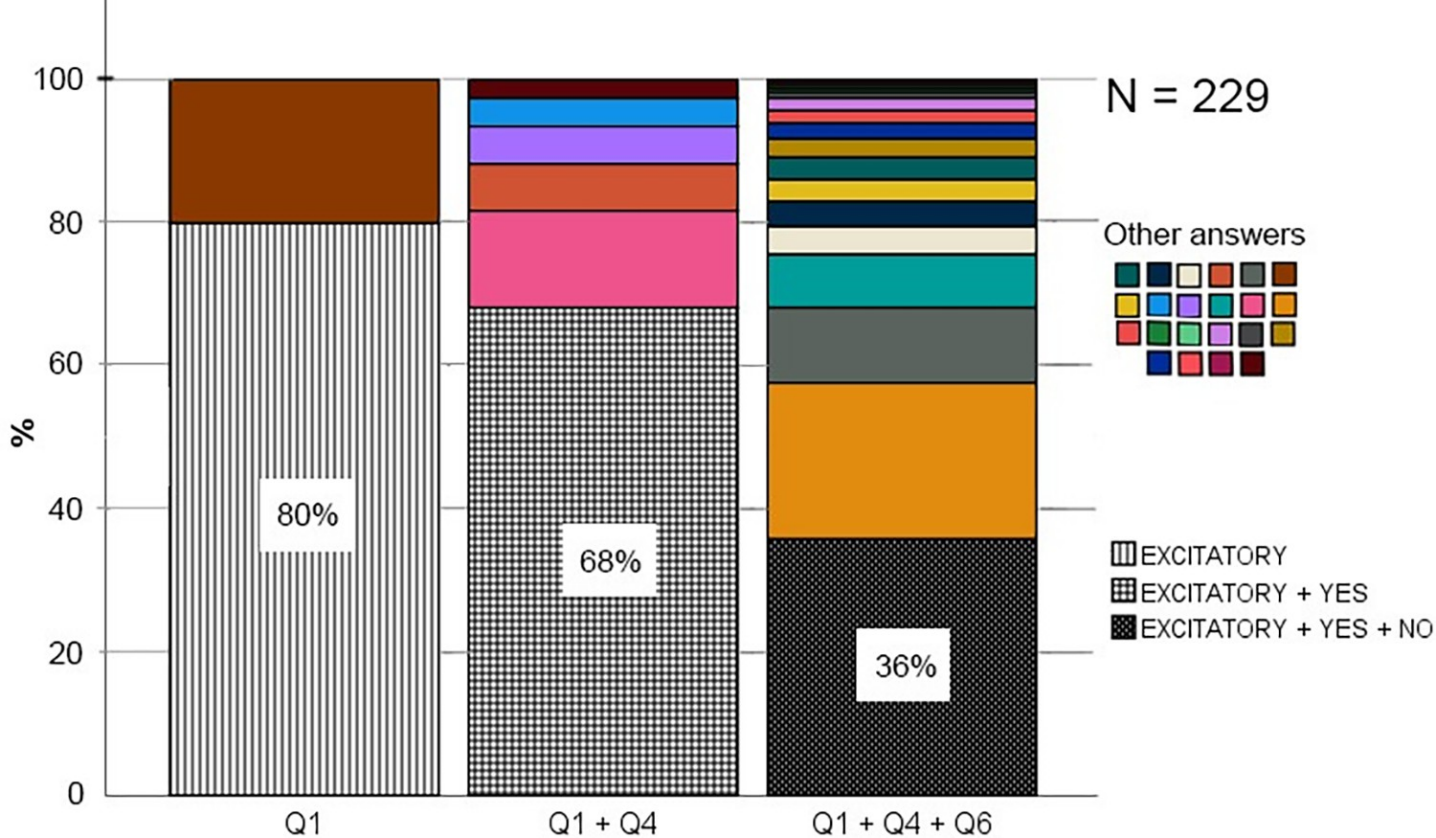
Stacked bar graphs showing the percentage of students within each conceptual category of synaptic action identified from their answers on question 1 (Q1), within each combination of answers on question 1 and 4 (Q1 + Q4), and within each combination of answers to question 1, 4, and 6 (Q1 + Q4 + Q6). Only the category EXCITATORY is specified for Q1, only the category YES is specified for Q4, and only the category NO is specified for Q6. This is because they are the only relevant categories for the purpose of demonstrating the prevalence and persistence of the Excitatory and the Exclusive Excitatory conception.

To investigate students’ conceptions of synaptic action, they were initially given an open-ended question asking about what a nerve cell, which receives a strong nerve signal, does with the signal (see question 1 for details). The student’s answers were quite uniform (see Table 1 for examples), and therefore made up only two categories: “Excitatory” and “Don’t know”. The categories were deductively generated with an inter-coder reliability of α = 0.9559, demonstrating that the three coders did agree. To determine the prevalence of the Excitatory conception, the percentage was calculated, and as shown in Fig 5, most students provided an answer consistent with a conception of synaptic action as excitatory (80%). The remaining students answered that they did not now, or provided an answer which was interpreted as if they did not know (20%). Importantly, no students answered in line with an inhibitory, or modulatory conception, and therefore no students demonstrated the correct understanding of synaptic action as either excitatory or inhibitory. Clearly, the Excitatory conception was much higher than expected from a random distribution (25%) between the four relevant conceptual categories (excitatory, inhibitory, either excitatory or inhibitory, and modulatory). Therefore, based on its’ relative prevalence, the Excitatory conception is considered prevalent, and it is the only prevalent conception of synaptic action in this sample of students. To determine the persistence of this Excitatory conception, the students were subsequently asked two more questions which directly confronted them with their conception on this aspect. The first question asked whether a neuron, which sends a nerve impulse, can stimulate another neuron to send a nerve impulse (question 4). As shown in Fig 6, this was confirmed by most of the students who answered “Excitatory” on question 1. Using a chi-square test followed by a post-hoc, the study further demonstrated that this proportion (i.e., the proportion of students answering “Excitatory” on question 1 and “Yes” on question 4) was significantly higher than expected by chance: *X*^*2*^ (1, *N* = 229) = 0.846, *p* = 0.0036 (S3 Table). Therefore, the conclusion is that the Excitatory conception is persistent over the course of an open-ended question and a multiple-choice question. Since the conception of synaptic action as excitatory is not incorrect per se, just incomplete, a second question was necessary to determine if their conception was exclusively excitatory, because that would be incorrect. Therefore, the second question asked whether a neuron, which sends a nerve impulse, can prevent another neuron from sending a nerve impulse (question 6). As shown in Fig 6, this was refuted by most of the students who answered “Excitatory” on question 1 and “Yes” on question 4, and therefore confirming the Exclusively Excitatory conception. Again, using a chi-square test followed by a post-hoc demonstrated that also this proportion (i.e., the proportion of students answering “Excitatory” on question 1, “Yes” on question 4, and “No” on question 6), was significantly higher than expected by chance: *X*^*2*^ (1, *N* = 229) = 4.36, *p* = 0.037 (S4 Table). Therefore, the study concludes that the Exclusively Excitatory conception is persistent over the course of three questions, two different types (open-ended and multiple-choice). In summary, these results show that most students seem to have a clear conception of synaptic action as excitatory, and for a significant proportion of students, this conception was exclusively excitatory. This conception was prevalent, and it showed some persistence.

**Table 1:** Examples from the coding of students’ answers to question 1: A nerve cell sends a strong nerve signal to another nerve cell. What does the other nerve cell do with the signal (what happens to the signal)?.

Stud nr	Students’ responses	Coded category (coder 1)	Coded category (coder 2)	Coded category (coder 3)
1	Send it up to the brain	Excitatory	Excitatory	Excitatory
2	Interpret the signal and pass it on	Excitatory	Excitatory	Excitatory
3	Pass it on to the brain	Excitatory	Excitatory	Excitatory
4	Perceives the signal	Don’t know	Don’t know	Don’t know
5	The signal is passed on to the brain	Excitatory	Excitatory	Excitatory
6	The signal is first passed on to the spinal cord and then to the brain which interprets the signal and then to the nerve cell again.	Excitatory	Excitatory	Excitatory
7	It goes to the brain and warns the nerve cells	Excitatory	Excitatory	Excitatory
8	The signal is passed on or executed	Excitatory	Excitatory	Excitatory
9	The signal is passed further on to the brain	Excitatory	Excitatory	Excitatory
10	The nerve cell receives the signal and is taken up by the dendrite	Don’t know	Don’t know	Excitatory
11	Either the signal is passed on to a new nerve cell or to a muscle which contracts or stretches	Excitatory	Don’t know	Don’t know
…	Continues …	…	…	…

### What conceptions do students have of nerve signal origin?

**Fig 7:**
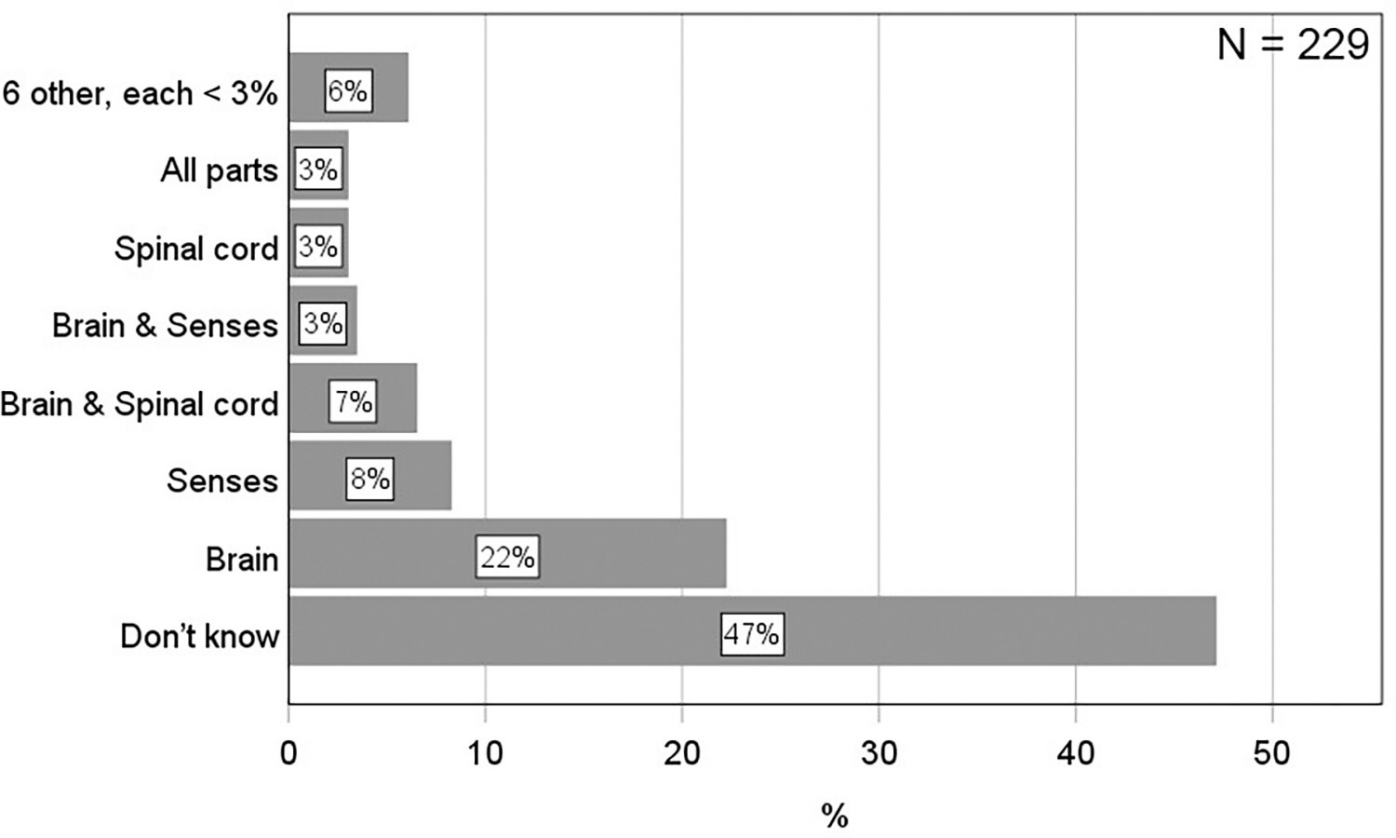
Bar graphs showing the percentage of students within each conceptual category of nerve signal origin identified from their answers on question 2 (see Fig 1 for details on question 2).

**Fig 8:**
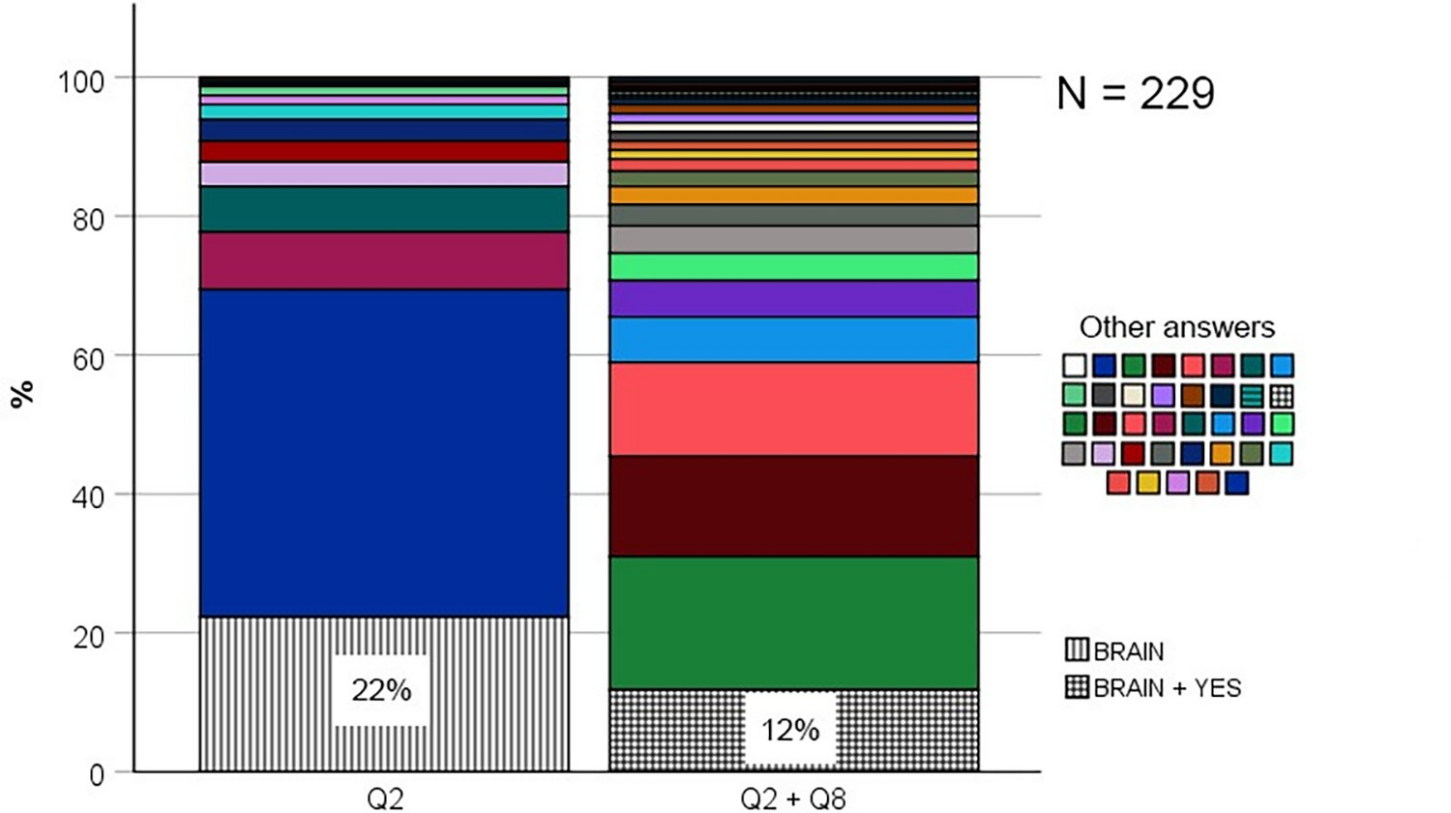
Stacked bar graphs showing the percentage of students within each conceptual category of nerve signal origin identified from their answers on question 2 (Q2), and within each combination of answers on question 2 and 8 (Q2 + Q8). Only the category BRAIN is specified for Q2, and only the category YES is specified for Q8. This is because they are relevant and sufficient for the purpose of demonstrating the prevalence and persistence of the BRAIN conception.

To reveal students’ conceptions of nerve signal origin, they were initially given an open-ended question asking about which part(s) of the nervous system can start nerve impulses independent of other nerve cells (see question 2 for details). The answers were categorized into twelve conceptions without any coding. Some of these conceptions consisted of only one single part of the nervous system, whereas others consisted of two or more. To determine the prevalence of each conception, the percentages were calculated (Fig 7). This showed that the most prevalent conception was the brain (22%), and this conception was almost three times more prevalent than the senses (8%) which was second most prevalent. The third most prevalent conception consisted of two parts of the nervous system: the brain and the spinal cord (7%). Surprisingly, the correct conception, which includes both the brain and the senses, had a prevalence of only 3%. The spinal cord and the all-parts categories also had a prevalence of 3%. The other six categories each represented less than 3% of the students and therefore collected in a category termed “Other”. The remaining and largest proportion of students reported that they did not know (47%). Using a binomial test, the study showed that the only conception which was significantly more prevalent than expected from a random distribution of students among the twelve identified categories (8.3%), was the brain-conception (22.3%, *p* < .001) (S5 Table). Therefore, based on its’ relative prevalence, the conclusion is that the brain conception is prevalent, and it is the only prevalent conception of nerve signal origin. To determine the persistence of the brain conception, the students were asked two more questions which directly confronted them with this conception. The first question asked whether a nerve impulse can start in the central nervous system (brain and spinal cord) without activating the senses first (question 8). As shown in Fig 8, this was confirmed by just above half of the students who answered BRAIN on question 2 (12%). However, using a chi-square test followed by a post-hoc showed that this proportion (i.e., the proportion of students answering the BRAIN on question 2 and YES on question 8) was not significantly higher than expected by chance *X*^*2*^ (1, *N* = 229) = 0.09, *p* = .76. Therefore, the conclusion is that the brain-conception is not persistent over the course of these two different types of questions (open-ended and multiple-choice). To check if the non-persistent brain conception was exclusively the brain, the second question asked whether a nerve impulse can start within the senses by stimulating the sensory cells with external stimuli (question 5). Answering NO on this question would support an exclusive brain conception. However, the results showed that the proportion of students answering BRAIN on question 2 and NO on question 5 was only 3.5% which is not significantly higher than expected by chance *X*^*2*^ (1, *N* = 229) = 0.01, *p* = .92. Therefore, the study conclude that the initial brain-conception was not exclusively the brain. In summary, these results show that almost half of the students seem to lack a clear conception of where the nerve signals originate from. However, those who have a conception can be grouped into one of twelve conceptual categories, where the brain-conception is most prevalent. However, it is not persistent. Nor is it an exclusive brain-conception.

### How do students’ conceptions of neural circuit architecture, synaptic action, and nerve signal origin influence their understanding of the system level?

**Fig 9:**
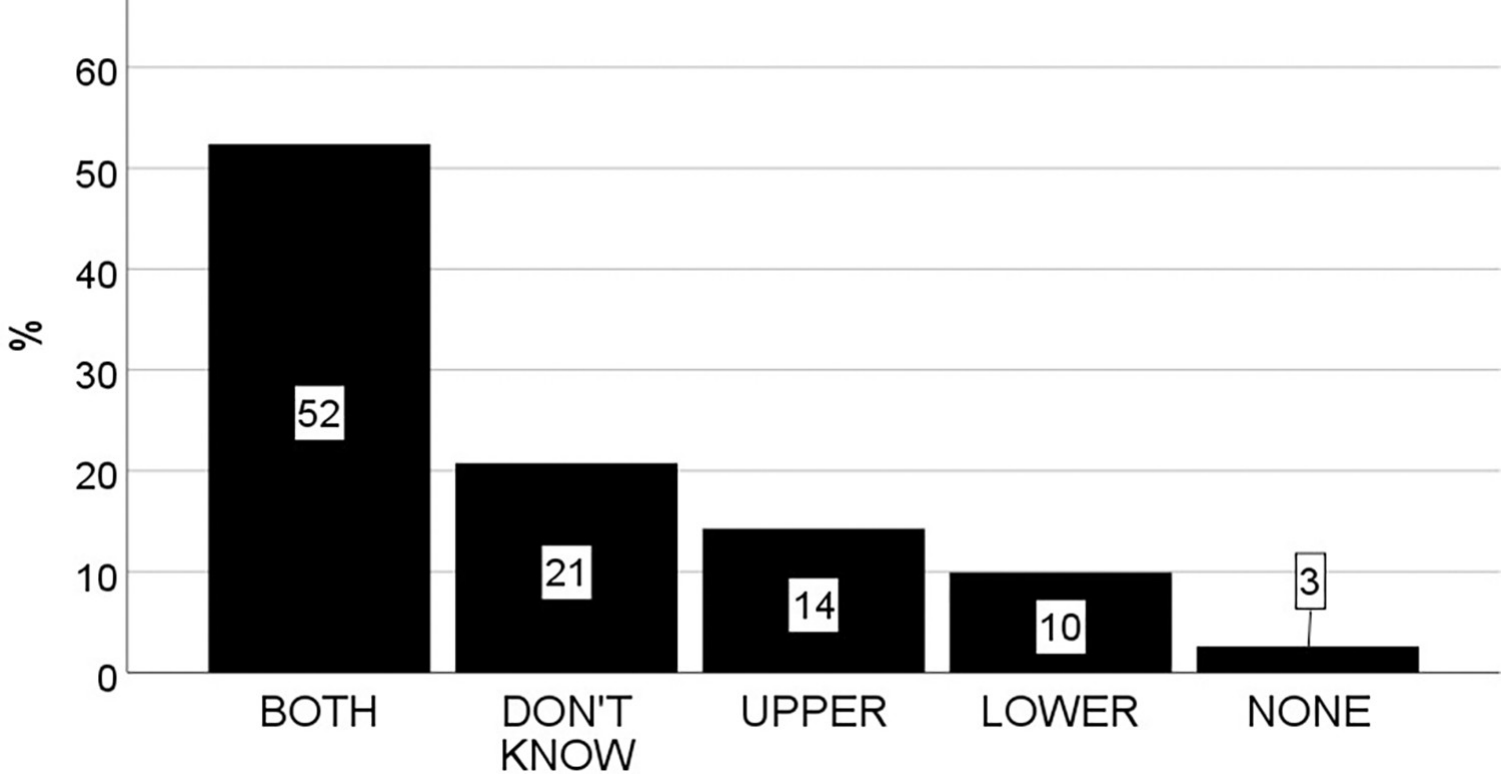
Bar graphs showing the percentage of students within each response alternative on question 9 (see Fig 1 for details on question 9).

To reveal how students’ conceptions of neural circuit architecture, synaptic action, and nerve signal origin influence their understanding of the system level, they received a drawing of a simple neural circuit (Fig 1, question 9), and they were asked if they could determine the output from a given input, i.e., which eye muscle(s) receive(s) signal if a nerve signal is sent from the sensory nerve cell in the circuit? As shown in Fig 9, only a minority checked the correct answer which is “None” (3%). Most students answered “both” muscles (52%). Many students did not know (21%), and a few students answered “upper” or “lower”. These results clearly demonstrate that most students have an incorrect understanding of the system, and this is consistent with the finding that most students had an incorrect conception, or lacking conception of one or more of the three investigated aspects. However, to casually link their understanding of the system to their conceptions of the three aspects, they were asked to add a comment to their answers. Although this resulted in only nine useful comments, they show that students’ conceptions of all three aspects, correct or incorrect, were used to explain their answers. In addition, students with a correct understanding of the system level demonstrated correct conceptions of all three aspects, and students with an incorrect understanding of the system demonstrated incorrect conception of one or more aspect (Table 2). For example, one student incorrectly answering the UPPER MUSCLE, explained that: “It sends the nerve signal further the green “path” because there is an inhibited neuron there” (stud nr 2). This comment was interpreted as follows: “the sensory neuron sends the nerve signal through the upper chain of neurons (which is green) because the lower chain has an inhibitory neuron”. This student seems to believe, incorrectly, that the nerve signal stops within the inhibitory neuron instead of correctly believing that the inhibitory neuron sends an inhibitory nerve signal that inhibits its’ target neurons. Therefore, regardless of having a text stating that the red cell sends inhibitory signals, this student seems unable to comprehend or use that information to revise his/her apparent incorrect conception of synaptic action as exclusively excitatory. This provides additional evidence for the persistence of the Excitatory conception. The student’s explanation also demonstrates an incorrect conception of neural circuit architecture. He/she did not notice or understand the diverging axon of the second excitatory neuron, which also makes a “green path” to the lower muscle (not just to the upper muscle). Therefore, regardless of having an image illustrating divergence, this student seems unable to comprehend or use the information to revise his/her apparent incorrect conception of neural circuit architecture as a chain of neurons connected one after another. This provides additional evidence for the persistence of the Chain conception. Finally, the student’s explanation also demonstrates a conception of the nerve signal as originating from the senses, which is correct in this context. In fact, the same was observed for all nine students who provided comments. The brain was not mentioned. This suggests that many students were able to comprehend and correctly use the information about nerve signal origin given in the text even though the selected answer at the system level was incorrect. This provides additional evidence for the lack of persistence of the Brain conception. The causal relationship between students’ conceptions of the single aspects and their understanding of the system was also demonstrated for the students who answered correct. For example, one student correctly answering NONE of the muscles, correctly explained that: “None of the muscles, because there are inhibitory nerve signals which “disturbs”, namely inhibits/damages, the signal so that it does not reach the eye muscles” (stud nr 8). This student clearly shows a correct conception of the two aspects (synaptic action and neural circuit architecture) which the other student has an incorrect conception of. He/she points out that there are inhibitory nerve signals, and that these signals negatively impact both pathways (through divergence). In summary, these results demonstrate that most students’ understanding of the system is incorrect, and that the Open Chain conception of neural circuit architecture as well as the Exclusively Excitatory conception of synaptic action contribute to this incorrect understanding. The Brain conception of nerve signal origin, however, could not be casually linked to an incorrect understanding because all nine students providing comments, both those with a correct answer and those with an incorrect answer, seemed to understand that the nerve signal originated from the sensory neuron in this particular context.

**Table 2:** Relationships between students’ understanding of the system and their conceptions of the three basic aspects: 1) Neural circuit architecture, 2) Synaptic action, and 3) Nerve signal origin.

Stud nr	Students’ understanding of the system			Basic aspects which are misconceived	Students’ specific alternative conceptions of basic aspects
	Students’ answer on question 9	Students’ comments	Interpretation of students’ comments		
1	Upper (wrong)	I think it is the upper because if you see the sensory nerve cell, it shows a green nerve cell where one of them leads to a green and the other to a red. Since the red are inhibit, I don’t think they can send signals further on.	1) The student ignores the divergent connection of the second order excitatory neuron (green).	1) Neural circuit architecture	Chain of neurons connected one after another.
			2) The student thinks that the nerve signal stops within the inhibitory neuron.	2) Synaptic action (inhibition)	The inhibitory neuron is inhibited
			3) The student thinks the nerve signal originates from the sensory nerve cell.		
2	Upper (wrong)	It sends the nerve signal further the green “path” because there is an inhibited neuron there	1) The student ignores the divergent connection of the second order excitatory neuron (green).	1) Neural circuit architecture	Chain of neurons connected one after another.
			2) The student thinks that the nerve signal stops within the inhibitory neuron.	2) Synaptic action (inhibition)	The inhibitory neuron is inhibited
			3) The student thinks the nerve signal originates from the sensory nerve cell.		
3	Upper (wrong)	It has a completely green pathway to the eye.	1) The student ignores the divergent connection of both second order neurons.	1) Neural circuit architecture	Chain of neurons connected one after another.
			2) The student thinks that the inhibitory neuron inhibits signal transmission.		
			3) The student thinks the nerve signal originates from the sensory nerve cell.		
4	Lower (wrong)	I think lower eye muscle because upper eye muscle must go through three synapses, but lower has to go through only two, and then I think that it goes faster to the lower muscles.	1) The student acknowledges the divergent connection of the second order excitatory neuron (green).		
			2) The student ignores inhibition or thinks that the nerve signal stops within the inhibitory neuron.	2)Synaptic action (inhibition)	The inhibitory neuron is inhibited, or the red neuron is excitatory
			3) The student thinks the nerve signal originates from the sensory nerve cell.		
5	Both (wrong)	In the drawing one can see that it sends signals to both eye muscles	1) The student acknowledges the divergent connection of the second order excitatory neuron (green).		
			2) The student ignores inhibition or thinks that the nerve signal stops within the inhibitory neuron.	1) Synaptic action (inhibition)	The inhibitory neuron is inhibited, or the red neuron is excitatory
			3) The student thinks the nerve signal originates from the sensory nerve cell.		
6	Both (wrong)	The signal stops within the inhibitory neuron	1) The student acknowledges the divergent connection of the second order excitatory neuron (green).		
			2) The student thinks that the nerve signal stops within the inhibitory neuron	1) Synaptic action (inhibition)	The inhibitory neuron is inhibited
			3) The student thinks the nerve signal originates from the sensory nerve cell.		
7	Both (wrong)	It blocks none because it blocks just one of the inflows, not both	1) The student acknowledges the divergent connections.		
			2) The student thinks that the nerve signal stops within the inhibitory neuron	2)Synaptic action (inhibition)	The inhibitory neuron is inhibited
			3) The student thinks the nerve signal originates from the sensory nerve cell.		
8	None (correct)	None of the muscles because there are inhibitory nerve signals which “disturbs”, namely inhibits/damages, the signal so that it does not reach the eye muscles.	1) The student acknowledges the divergent connections of both second order neurons.		
			2) The student thinks that the inhibitory neuron inhibits signal transmission in both pathways (correct).		
			3) The student thinks the nerve signal originates from the sensory nerve cell.		
9	None (correct)	The red nerve cell deactivates the signal from the green, right?	1) The student acknowledges the divergent connections of both second order neurons.		
			2) The student thinks that the inhibitory neuron inhibits signal transmission in both pathways (correct).		
			3) The student thinks the nerve signal originates from the sensory nerve cell.		

## Discussion

The present study has shown that the most prevalent conception of neural circuit architecture is the Open Chain conception, the most prevalent conception of synaptic action is the Exclusively Excitatory conception, and the most prevalent conception of nerve signal origin is the Brain conception. In addition, the study has shown that the Open Chain conception and the Exclusively Excitatory conception prevent students from understanding the system. Therefore, these conceptions must be changed. Below, each conception will be discussed in the context of theories on conceptual learning with the goal to propose adequate instructional strategies for conceptual change.

### The Open chain conception and its’ instructional implications

For aspect 1) neural circuit architecture, eight different conceptions were identified, of which the Open Chain was the major conception. Since this conception differs from what is known to be scientifically correct, and since it was found to be prevalent and showed some persistence, it seems to comply with the definition of an alternative conception [33,34]. Furthermore, since the students already had been exposed to school science on the topic, it is reasonable to assume that the Open chain conception partly stems from instruction. This is supported by the fact that the Open chain can be used to successfully explain the simplified knee-jerk reflex illustrated in the student’s science textbooks [e.g., 22–24] which is commonly used to teach neural circuit architecture at this educational level. These books present the knee-jerk reflex with only two neurons connected in an open chain from the sensory organ to the muscle. Thus, the Open chain conception does have some explanatory power, and may therefore be further categorized as an alternative conception of the synthetic type, in line with [38]’s framework theory. Synthetic conceptions, in contrast to fragmented conceptions, can be used to explain certain phenomena, or certain aspects of phenomena, but their explanatory power is limited. Consistent with this limitation, the present study showed that the Open chain conception could not be used successfully to explain, or predict, the outcome of the neural circuit in question 9. Hence, this circuit was beyond the Open chain’s explanatory power. Therefore, in the context of the neural circuit in question 9, the Open chain conception may be further categorized as an alternative synthetic conception with an Overgeneralized Domain of Validity, in line with both [38]’s Framework theory and [39]’s Domain of Validity theory (DoV). This means that the Open chain concept was applied in a domain (context) where it was inappropriate, i. e. applied beyond its’ domain of validity. In this case, the inappropriate domain of validity was the neural circuit in question 9. Therefore, according to the DoV, instructional design should focus on reducing the concept’s domain of validity. This can be achieved by making the limitations of the Open chain conception explicit for the students, for example by introducing a cognitive conflict. In this way the students learn that the Open chain conception can only explain simplified neural circuits (e. g. the simplified version of the knee-jerk circuit), and that other conceptions are needed to explain more realistic neural circuits. If more realistic neural circuits are introduced, then a scientific conception must be taught, and students would have to undergo a conceptual change from the Open chain conception to the Network conception. According to the Framework theory, this would require ontological and epistemological restructuring of the student’s knowledge system. However, since the Open chain and the Network conceptions belong to the same basic ontological category, called entity, the only necessary restructuring may be epistemological. To achieve this, instruction and curricula should, according to [38], provide all the information necessary for such restructuring, including textbook language and illustrations that are accurate. From an anatomical point of view, this may simply mean adding a few more branches to the textbook illustrations of neurons, some on the input side (dendrites) and some on the output side (branches on the axon). However, from a physiological point of view, the situation may be more complex since students’ understanding must change from a single-line serial (chain) propagation of nerve signals (single dimension) to a multiple parallel-lines input-output propagation (multiple dimensions). This may have additional epistemological challenges that require more advanced forms of concretization.

### The exclusively excitatory conception and its’ instructional implications

For aspect 2) synaptic action, only one conception was identified. This was the Excitatory conception, and for a significant proportion of students this meant exclusively excitatory. Since also this conception differs from what is known to be scientifically correct, and since it was both prevalent and showed some persistence, it seems to comply with the definition of an alternative conception [33,34]. Furthermore, since the students already had been exposed to school science on the topic, it is reasonable to assume that also the Excitatory conception partly stems from instruction. This is supported by the fact that the Excitatory conception can be used to successfully explain the simplified knee-jerk reflex illustrated in the student’s science textbooks [e.g., 22–24] which is commonly used to teach about synaptic action at this educational level. These books present the knee-jerk reflex with only a single synapse which is excitatory. Thus, the Excitatory conception does have some explanatory power, and may therefore be further categorized as an alternative conception of the synthetic type, in line with [38]’s framework theory. Consistent with the limited explanatory power of synthetic conceptions, the present study showed that the Excitatory conception could not be used successfully to explain, or predict, the outcome of the neural circuit in question 9, i. e. the circuit was beyond the Excitatory conception’s explanatory power. This suggests that the Excitatory conception was applied in a context where it was not appropriate, and that the inappropriate context was the neural circuit in question 9. Therefore, also the Excitatory conception may be further categorized as an alternative synthetic conception with an Overgeneralized Domain of Validity, in line with [39]’s DoV. Consequently, instructional design should focus on reducing the Excitatory conception’s domain of validity. This can be accomplished by making the limitations of the Excitatory conception explicit for the students as described for the Open chain conception above. It should also be clear that if a more realistic neural circuit is introduced, then also a more scientific conception must be taught, and students would have to undergo a conceptual change from the excitatory conception to the excitatory/inhibitory conception. According to the Framework theory, this would require an ontological and epistemological restructuring of the student’s knowledge system. However, since the basic ontological category for the two concepts is similar (process), the required restructuring may be limited to epistemology. In addition, since the type of ontological category is “process”, the required restructuring only involves physiology, not anatomy. Therefore, in line with [37], instruction and teaching material only need to provide information necessary for an epistemological restructuring related to physiology. This may be achieved through textbook language and illustrations that are accurate. However, scientific processes (in this case physiology) are often difficult to learn with the use of static teaching material. Therefore, the epistemology on inhibitory action, and particularly the different consequences of inhibitory and excitatory actions in a neural circuit, may require more advanced forms of concretization material like dynamical models. It should also be noted that in secondary school, it probably makes more sense to learn excitatory and inhibitory action at the neuron-level than at the synapse-level [1]. A neuron usually has either an inhibitory synaptic action or an excitatory synaptic action on all its target cells, rarely both. In addition, neurons are bigger and more accessible for learning than their synapses. Therefore, concerning epistemology, it may be better to use the terms excitatory and inhibitory neurons, as suggested by [1], even though the action takes place at the synapse.

### The brain conception and its’ instructional implications

For aspect 3) nerve signal origin, the major conception appearing from the open-ended question was that the nerve signals originate from the “brain”. However, since it was not confirmed that this conception was exclusively the brain, this is not wrong, just incomplete. A complete conception would include both the brain [21] and the senses [20]. In addition, although the brain conception was prevalent, it was not persistent. Therefore, it does not seem to comply with the definition of an alternative conception. Rather, its’ low persistence suggests that it was more spontaneously triggered by the specific context of open-ended question 2. Therefore, the brain conception may be the result of a (yet unknown) p-prim, in line with [36]’s Knowledge in Pieces theory (KiP), and not the result of school science. This is supported by the fact that student’s science textbooks usually present the sensory origin of nerve signal, not the brain origin [e.g., 22–24]. It also seems to comply with the instruction of teachers, since the sensory origin (exclusively sensory) is a common alternative conception among school teachers [53,54]. A potential p-prim triggering the Brain conception of nerve signal origin may be something like the Ohm’s p-prim. This is because the Ohm’s p-prim involves an Agent which is the locus of an Impetus [40], and because agency in humans is strongly associated with (free) will. Applying the Ohm’s p-prim to the nervous system would therefore likely result in ascribing agency to the brain rather than to the senses, and impetus to the nerve signal. Since p-prims only work in some contexts but not in others, i. e. only when used inside their range of legitimate applicability [40], the KiP theory suggest that instruction should expose students to multiple contexts, while encouraging student reflection under guidance. In this way, students may find contexts where the p-prims are productive and where they are not. This is very similar to the DoV theory which claims that alternative conceptions often work in some contexts but not in others, i. e. only when used inside their domain of validity [39]. The instructional implications are also quite similar for the two theories, both highliting the importance of the context associated with the conceptions. However, whereas the KiP theory talks about conceptual refinement, and is vague on how to deal with the context during instruction, the DoV theory talks about conceptual change through cognitive conflict, and it emphasizes that the context should be made explicit to the students together with the concept. For the present study, this means that instruction should make explicit to the students which contexts the brain conception and the sensory conception are appropriate. Contexts in this topic means neural circuits. Unfortunately, there are only two neural circuits dominating the current science literature for this educational level (the knee jerk circuit and the withdrawal circuit), and in none of them does the brain conception apply. Thus, the number and types of available neural circuits for this educational level must be increased if students are expected to obtain a correct/complete conception of nerve signal origin and develop a proper understanding of the nervous system at the system level.

### The influence of students’ conceptions on their understanding of the system level, and its’ instructional implications

The study showed that students’ alternative conceptions of two aspects (neural circuit architecture and synaptic action) prevented them from understanding the system. However, students’ conceptions of all three aspects, correct or incorrect, were used to explain their answers on question 9, and since this demonstrated that students with correct explanations (correct conceptions) of all three aspects checked the correct answer (correct understanding of the system level), and students with incorrect explanation (conception) of one or more aspect checked an incorrect answer (incorrect understanding of the system), it is very likely that all three aspects are important for understanding the system level. Furthermore, since the aspects were identified by applying the systems thinking approach of Arnold and Wade [15] to the system level of the nervous system [13,14], the study provides empirical support to the systems thinking approach: i. e. it is a suitable tool for identifying content knowledge important for understanding the system level of the nervous system. Consequently, the approach may also be used to successfully identify content knowledge comprising the remaining six elements of the systems thinking approach. Such content knowledge may be important to understand other neural circuits than the one presented in question 9, or the knee-jerk reflex, or the withdrawal reflex. As such, the systems thinking approach may be a promising tool for future research and development of instruction on the nervous system.

### Alternative conceptions—a faulty education, or an early stage in an educational progression?

The finding that students don’t understand the system level of the nervous system, and that this is caused by their alternative conceptions or lack scientific conceptions, of neural circuit architecture, synaptic action, and nerve signal origin, appears problematic. However, is this a consequence of faulty education, or an early stage in a research-based educational progression? An example of the latter is that the Open chain and the Excitatory alternative conceptions are taught in lower secondary school, and the Network and the Inhibitory conceptions in upper secondary. This could be supported by the framework theory [38] which says that conceptual learning in school progresses through alternative conceptions like fragmented and/or synthetic conceptions before reaching the scientific conception. To some extent, such a progression may also find support in Piaget’s cognitive developmental stage theory which divides students’ cognitive development into four stages, and where only the last stage (the formal operational stage) allows for comprehensive understanding [55]. If the students have not yet entered the last stage, one may argue that their thinking abilities are not yet sufficiently developed to allow for understanding the concepts of inhibitory synaptic action and network architecture of neural circuits. However, students usually enter the last stage at the age of 11–12 years old [55], and most students in the present study were 14–15 years old. Therefore, only a minority of the students, if any, would likely be limited by cognitive development. In addition, it is not even clear if the formal operational stage is required for acquiring the scientific conceptions of synaptic action and neural circuit architecture. Therefore, educational theory does not provide much support for implementing such a progression in education. Furthermore, if such a progression did exist, it should be specified in the curricula. However, the Norwegian curriculum states that students, after completing lower secondary education, should be able to describe how drugs, medicine, environmental toxins, and doping influence the nervous system [12]. Since these substances impact the nervous system through both excitatory and inhibitory synapses [e.g. 56], the national curriculum does not specify such a progression. Likewise, the US curriculum states that “connections of components in a system” as well as “information flow both within and between systems” should be taught [11]. In fact, “connections of components” is already mentioned in fourth and fifth grade, but there it is phrased as “components and their interactions”. Since their (neurons) interactions include both excitatory and inhibitory synaptic actions, this demonstrates that also the USA curriculum has not specified such a progression. Finally, such a progression in Norway and other countries in Europe is problematic because lower secondary education is often the last formal education on the nervous system. Therefore, these students’ conceptions of the topic likely represent the conceptions of most people in society. The question then, is whether the simplified knee-jerk reflex, which is the Domain of Validity for the two alternative conceptions identified in the present study, represents a sufficient level of understanding? The consequences would be that peoples’ understanding of how the nervous system contributes to behavior, thoughts, and feelings, is only as a simple feed-forward, input-output machine with no processing capacity. Such an understanding is clearly incompatible with complex behavior, and may force people to seek other systems, possibly other disciplines, which in the worst case are not scientific, to explain their physical behavior and mental states. This is problematic in a time where both physical and mental health has gained a central place in the curriculum [12]. Therefore, the alternative conceptions, or lack of scientific conceptions found in the present study, are likely not, and should not be, a consequence of an early stage in a research-based educational progression. Rather, it is more likely to be a consequence of faulty education. The apparent discrepancy between curricula and textbooks for example, suggests that textbooks are inadequate. Inadequate textbooks further suggest that instruction is inadequate. The consequence is that critical aspects necessary for understanding the system level are not properly taught. Textbook authors and teachers may not even be aware of these aspects since the present study seems to be the first one trying to identify them. Therefore, applying a systems-thinking approach combined with updated textbooks would be a good start to improve education on the topic. Next, hands-on learning material that can properly concretize the three aspects, may be another promising strategy for future teaching.

### Limitations of the study

Limitations of the present study mainly concern the questionary. At least three issues may question the validity of the results. One issue concerns the amount and type of information provided in the multiple-choice items which may have provided cues to the right answer. This includes, for example, item 8 which tried to verify the brain conception of nerve signal origin initially identified in open-ended question 2. Another example is item 5 which tried to verify if the brain conception was exclusively the brain. Too much information in these items may perhaps explain the low persistence of the brain conception.

A second issue concerns the number and types of multiple-choice items which may have been insufficient for an adequate identification of students’ conceptions. This includes, for example, the aspect of neural circuit architecture which were probed with only a single multiple-choice item (item 7), whereas the two other aspects were probed with two. However, the reason for only including one multiple-choice item for the aspect of neural circuit architecture was that it was sufficient to claim that it was incorrect, whereas the two other aspects required two multiple-choice items to claim that. In addition, it was difficult to make another item without providing too much information. According to [45], the risk of providing cues is, unfortunately, a common and unavoidable concern of multiple-choice test.

A third issue concerns the small size of the questionary which, of course, limits how deep one can probe into students’ conceptions. Greater depth could be achieved by interviews, but since the study had clear hypotheses from the literature (students’ science textbooks), a questionary with a mix of open and closed items was preferred. Although there is an arsenal of tools developed with the purpose of probing into students’ alternative conceptions, each study needs to carefully balance the number of aspects to investigate with the number of questions needed to identify alternative conceptions within the aspects. This is particularly important when working with students at these young ages, since their motivation to properly complete the questions may quickly fade. The present study applied some of these tools and provides a first glimpse into lower secondary student’s conceptions on the topic.

### Implications and future prospects

Since the nervous system is the only source of thoughts, feelings, and complex behavior, and since the curriculum emphasize both physical and mental health more than ever before [12], the nervous system is clearly an important organ to understand. Furthermore, since the science curricula both nationally and internationally as well as research on the topic [1] emphasize an understanding of the system, and not just knowledge of isolated facts [10–12], students should presumably understand the system level. However, as the present study has demonstrated, an understanding of the system level as derived from [13–15], has not been reached, and this does not seem to be due to a research-based learning progression, but rather a faulty education. Therefore, the overall implication of this study is that teaching practice must change. This means, for example, that textbooks must be updated, teachers must be trained, and models of the nervous system must be improved, not at the level of the brain and single neurons, but at the level of neural circuits. Converging and diverging connections must be included. Excitatory and inhibitory neurons (synaptic actions) must be included, and both the sensory and the brain origin of nerve signals must be included. This may be accomplished by expanding the number and diversity of neural circuits in teaching material, for example like the Direction selective visual network in the retina [57], or perhaps even the swimming network of fish [58]. These circuits may not be familiar to the teaching community, so researchers would have to make them available along with many other circuits. Using a variety of neural circuits in the science classroom would broaden students’ understanding of the system level and more likely also facilitate their ability to transfer knowledge about the nervous system to other systems. Including neural circuits may also allow for more practical activities since their functions are diverse and varies in a way which is logically related to their architecture. Teachers may even use models to provide “hands on” activities and create multisensory and concrete experiences among the students. However, more details on how this can be done must be provided by future studies.

## Conclusion

The present study has shown that many students in lower secondary education have an immediate conception of the brain as the origin of nerve signals. In addition, many students hold the alternative conceptions that 1) neurons are connected in a chain, one single neuron after another, and 2) that synaptic action is exclusively excitatory. These alternative conceptions prevent students from understanding the nervous system at a level which is considered important by the curricula nationally and internationally. This suggests that current teaching on the system must change. Since similar curricula goals and textbook content exist in other countries, the present results may be representative across nations.

## Supporting information

S1 Table Descriptive and test statistics for a Binomial test comparing the proportion of students answering Chain (open) on question 3 with the expected proportion if the answers were randomly distributed between the eight conceptual categories. (PDF)

S2 Table. C hi-square test results and a crosstabulation of student’s responses to question 3 and 7, including data from a post hoc test (chi-square values and p-values for each combination of answers). (PDF)

S3 Table Chi-square test results and a crosstabulation of student’s responses to question 1 and 4, including data from a post hoc test (chi-square values and p-values for each combination of answers). (PDF)

S4 Table Chi-square test results and a crosstabulation of student’s responses to question 1, 4 and 6, including data from a post hoc test (chi-square values and p-values for each combination of answers). (PDF)

S5 Table Descriptive and test statistics for a Binomial test comparing the proportion of students answering Brain on question 2 with the expected proportion if the answers were randomly distributed between the twelve conceptual categories. (PDF)
