# Supplementary material for: Mapping lower secondary school students’ conceptions of three aspects critical for understanding the nervous system
Source: PLoS One. 2024 May 6;19(5):e0301090. doi: 10.1371/journal.pone.0301090 (PMC11073672; doi:10.1371/journal.pone.0301090)
Supplement: S1 Table — (PDF) [file pone.0301090.s001.pdf]

Table S1: Descriptive and test statistics for a Binomial test comparing the proportion of students answering Chain (open) on question 3 with the expected proportion if the answers were randomly distributed between the eight conceptual categories.

**Discriptive statistics**						
	N	Mean	Std. Dev.	Min	Max	
Binomial Question 3	229	0,34	0,473	0	1	
**Binomial test**						
		Category	N	Observed prop.	Test prop.	Exact Sig. (1-tailed)
Binomial Question 3	Chain (open)	1	77	0,336	0,125	<0,001
	The seven other conceptual categories	0	152	0,664		
	Total		229	1,000		
