# Supplementary material for: Mapping lower secondary school students’ conceptions of three aspects critical for understanding the nervous system
Source: PLoS One. 2024 May 6;19(5):e0301090. doi: 10.1371/journal.pone.0301090 (PMC11073672; doi:10.1371/journal.pone.0301090)
Supplement: S2 Table. C — (PDF) [file pone.0301090.s002.pdf]

Table S2: Chi-square test results and a crosstabulation of student's responses to question 3 and 7, including data from a post hoc test (chi-square values and p-values for each combination of answers).

**Chi-square test**						
	Value	df	Asymptotic Significance (2-sided)			
Pearson Chi-Square	29,77	16	0,0192			
Likelihood Ratio	31,78	16	0,0107			
N of Valid Cases	229					
**Crosstabulation**			Question 7			
			Don't know	No	Yes	Total
Question 3						
	Chain (open)	Count	9	6	62	77
		% within Question 3	11,69	7,79	80,52	100%
		Adjusted z values	-1,08	-3,12	3,40	
		Chi-square values	1,16	9,75	11,58	
		p-value	0,28182	0,00179	0,00067	
	Chain (closed)	Count	2	2	6	10
		% within Question 3	20	20	60	100%
		Adjusted z values	0,42	0,06	-0,37	
		Chi-square values	0,18	0,00	0,14	
		p-value	0,67169	0,94856	0,70819	
	Convergence	Count	1	3	1	5
		% within Question 3	20	60	20	100%
		Adjusted z values	0,30	2,34	-2,16	
		Chi-square values	0,09	5,48	4,68	
		p-value	0,76698	0,01926	0,03045	
	Divergence	Count	1	2	1	4
		% within Question 3	25	50	25	100%
		Adjusted z values	0,54	1,58	-1,72	
		Chi-square values	0,30	2,49	2,96	
		p-value	0,58588	0,11488	0,08559	
	Complex net	Count	0	0	4	4
		% within Question 3	0	0	100	100%
		Adjusted z values	-0,86	-0,98	1,46	
		Chi-square values	,73	,97	2,14	
		p-value	0,39144	0,32511	0,14312	
	Simple net	Count	1	2	1	4
		% within Question 3	25	50	25	100%
		Adjusted z values	0,54	1,58	-1,72	

		Chi-square values	0,30	2,49	2,96	
		p-value	0,58588	0,11488	0,08559	
	Bricks	Count	0	0	2	
		% within Question 3	0	0	100	100%
		Adjusted z values	-0,60	-0,69	1,03	
		Chi-square values	0,36	0,48	1,06	
		p-value	0,54629	0,48848	0,30262	
	Mesh	Count	1	1	0	2
		% within Question 3	50	50	0	100%
		Adjusted z values	1,37	1,11	-1,96	
		Chi-square values	1,88	1,23	3,83	
		p-value	0,17056	0,26703	0,05032	
	Don’t know	Count	20	28	73	121
		% within Question 3	16,5	23,1	60,3	100%
		Adjusted z values	0,55	1,60	-1,74	
		Chi-square values	0,31	2,55	3,04	
		p-value	0,57942	0,11041	0,08140	
	Total	Count	35	44	150	229
		% within Question 3	15,3	19,2	65,5	100%
