# Supplementary material for: Mapping lower secondary school students’ conceptions of three aspects critical for understanding the nervous system
Source: PLoS One. 2024 May 6;19(5):e0301090. doi: 10.1371/journal.pone.0301090 (PMC11073672; doi:10.1371/journal.pone.0301090)
Supplement: S3 Table — (PDF) [file pone.0301090.s003.pdf]

**Table S3:** Chi-square test results and a crosstabulation of student's responses to question 1 and 4, including data from a post hoc test (chi-square values and p-values for each combination of answers).

| **Chi-square test** | | | | | | |
|---|---|---|---|---|---|---|
| | Value | df | Asymptotic Significance (2-sided) | | | |
| Pearson Chi-Square | 10,191 | 2 | < 0,0061 | | | |
| Likelihood Ratio | 8,751 | 2 | < 0,0125 | | | |
| N of Valid Cases | 229 | | | | | |
| | | | | | | |
| **Crosstabulation** | | | Question 4 | | | |
| | | | Don't know | No | Yes | Total |
| Question 1 | | | | | | |
| | Excitatory | Count | 11 | 15 | 157 | 183 |
| | | % within Q3 | 6,0 | 8,2 | 85,8 | 100% |
| | | Adjusted z values | 2,91 | 1,02 | -2,91 | |
| | | Chi-square values | 8,47 | 1,04 | 8,47 | |
| | | p-value | 0,0036 | 0,3086 | 0,0036 | |
| | | | | | | |
| | Don't know | Count | 9 | 6 | 31 | 46 |
| | | % within Q3 | 19,6 | 13,0 | 67,4 | 100% |
| | | Adjusted z values | 2,91 | 1,02 | -2,91 | |
| | | Chi-square values | 8,47 | 1,04 | 8,47 | |
| | | p-value | 0,0036 | 0,3086 | 0,0036 | |
| | Total | Count | 20 | 21 | 188 | 229 |
| | | % within Q3 | 15,3 | 19,2 | 65,5 | 100% |
