# Supplementary material for: Mapping lower secondary school students’ conceptions of three aspects critical for understanding the nervous system
Source: PLoS One. 2024 May 6;19(5):e0301090. doi: 10.1371/journal.pone.0301090 (PMC11073672; doi:10.1371/journal.pone.0301090)
Supplement: S4 Table — (PDF) [file pone.0301090.s004.pdf]

Table S4: Chi-square test results and a crosstabulation of student's responses to question 1, 4 and 6, including data from a post hoc test (chi-square values and p-values for each combination of answers).

	**Chi-square test**			
Q1		Value	df	Asymptotic Significance (2-sided)
Don't know	Pearson Chi-Square	21,199	4	< 0,001
	Likelihood Ratio	20,875	4	< 0,001
	N of Valid Cases	46		
Excitatory	Pearson Chi-Square	13,19	4	< 0,010
	Likelihood Ratio	12,951	4	< 0,012
	N of Valid Cases	183		
Total	Pearson Chi-Square	29,905	4	< 0,001
	Likelihood Ratio	27,756	4	< 0,001
	N of Valid Cases	229		

**Crosstabulation**				Question 6			
				Don't know	No	Yes	Total
Question 1		Question 4					
	Excitatory	Don't know	Count	9	1	1	11
			% within Q3	81,8	9,1	9,1	100%
			Adjusted z values	3,4	-2,8	-0,6	
			Chi-square values	11,644	7,730	,40058	
			p-value	,00064	,0054	,5268	
		No	Count	4	7	4	15
			% within Q3	26,7	46,7	26,7	100%
			Adjusted z values	-0,7	-0,2	1,2	
			Chi-square values	,43580	,06120	1,43432	
			p-value	,509156	,804603	,231061	
		Yes	Count	50	83	24	157
			% within Q3	31,8	52,9	15,3	100%
			Adjusted z values	-1,8	2,1	-0,5	
			Chi-square values	3,25606	4,35674	,26020	
			p-value	,071160	,036863	,609981	

	Don’t know	Don’t know	Count	7	1	1	9
			% within Q3	77,8	11,1	11,1	100%
			Adjusted z values	3,4	-1,9	-1,4	
			Chi-square values	11,84535	3,68807	1,97340	
			p-value	,000578	,054803	,160087	
		No	Count	1	0	5	6
			% within Q3	16,7	0,0	83,3	100%
			Adjusted z values	-0,8	-2,1	3,0	
			Chi-square values	,61778	4,43572	9,11957	
			p-value	,431872	,035194	,002529	
		Yes	Count	6	17	8	
			% within Q3	19,4	54,8	25,8	100%
			Adjusted z values	-2,3	3,1	-1,0	
			Chi-square values	5,51240	9,84851	,96187	
			p-value	,018882	,001700	,326717	
		Total	Count	77	109	43	229
			% within Q3				100%
