# Supplementary material for: Mapping lower secondary school students’ conceptions of three aspects critical for understanding the nervous system
Source: PLoS One. 2024 May 6;19(5):e0301090. doi: 10.1371/journal.pone.0301090 (PMC11073672; doi:10.1371/journal.pone.0301090)
Supplement: S5 Table — (PDF) [file pone.0301090.s005.pdf]

Table S5: Descriptive and test statistics for a Binomial test comparing the proportion of students answering Brain on question 2 with the expected proportion if the answers were randomly distributed between the twelve conceptual categories.

**Discriptive statistics**						
	N	Mean	Std. Dev.	Min	Max	
Binomial Question 2	229	0,22	0,417	0	1	
**Binomial test**						
		Category	N	Observed prop.	Test prop.	Exact Sig. (1-tailed)
Binomial Question 2	Brain	1	51	0,223	0,083	<.001
	All other conceptions	0	178	0,777		
	Total		229	1,000		
